# Magnetic Resonance Imaging Features of Rhino-Orbito-Cerebral Mucormycosis in Post-COVID-19 Patients: Radio-Pathological Correlation

**DOI:** 10.3390/diagnostics13091546

**Published:** 2023-04-25

**Authors:** Rania Mostafa Hassan, Yassir Edrees Almalki, Mohammad Abd Alkhalik Basha, Mai Ahmed Gobran, Saad Misfer Alqahtani, Abdullah M. Assiri, Saeed Alqahtani, Sharifa Khalid Alduraibi, Mervat Aboualkheir, Ziyad A. Almushayti, Asim S. Aldhilan, Sameh Abdelaziz Aly, Asmaa A. Alshamy

**Affiliations:** 1 Department of Diagnostic Radiology, Faculty of Human Medicine, Zagazig University, Zagazig 44519, Egypt; raniahassan@medicine.zu.edu.eg (R.M.H.); mohammad_basha76@yahoo.com (M.A.A.B.); aashami@medicine.zu.edu.eg (A.A.A.); 2 Division of Radiology, Department of Internal Medicine, Medical College, Najran University, Najran 61441, Saudi Arabia; 3 Department of Surgical Pathology, Faculty of Human Medicine, Zagazig University, Zagazig 44519, Egypt; magibran@medicine.zu.edu.eg; 4 Department of Pathology, College of Medicine, Najran University Hospital, Najran University, Najran 61441, Saudi Arabia; smaalqahtany@nu.edu.sa; 5 Department of Surgery, College of Medicine, Najran University, Najran 61441, Saudi Arabia; aamodrba@nu.edu.sa (A.M.A.); alhafezsaeed@gmail.com (S.A.); 6 Department of Radiology, College of Medicine, Qassim University, Buraidah 52571, Saudi Arabia; salduraibi@qu.edu.sa (S.K.A.); a.aldhilan@qu.edu.sa (A.S.A.); 7 Department of Radiology and Medical Imaging, College of Medicine, Taibah University, Madinah 42353, Saudi Arabia; maboualkheir@taibahu.edu.sa; 8 Department of Diagnostic Radiology, Faculty of Human Medicine, Benha University, Benha 13511, Egypt; drsamehaly75@gmail.com

**Keywords:** MRI, mucormycosis, sinus, orbit, COVID-19

## Abstract

There has been a notable increase in rhino-orbito-cerebral mucormycosis (ROCM) post-coronavirus disease 2019 (COVID-19), which is an invasive fungal infection with a fatal outcome. Magnetic resonance imaging (MRI) is a valuable tool for early diagnosis of ROCM and assists in the proper management of these cases. This study aimed to describe the characteristic MRI findings of ROCM in post-COVID-19 patients to help in the early diagnosis and management of these patients. This retrospective descriptive study was conducted at a single hospital and included 52 patients with COVID-19 and a histopathologically proven ROCM infection who were referred for an MRI of the paranasal sinuses (PNS) due to sino-orbital manifestations. Two radiologists reviewed all the MR images in consensus. The diagnosis was confirmed by histopathological examination. The maxillary sinus was the most commonly affected PNS (96.2%). In most patients (57.7%), multiple sinuses were involved with the black turbinate sign on postcontrast images. Extrasinus was evident in 43 patients with orbital involvement. The pterygopalatine fossa was involved in four patients. Three patients had cavernous sinus extension, two had pachymeningeal enhancement, and one had epidural collection. The alveolar margin was affected in two patients, and five patients had an extension to the cheek. The awareness of radiologists by the characteristic MRI features of ROCM in post-COVID-19 patients helps in early detection, early proper management, and prevention of morbid complications.

## 1. Introduction

Severe acute respiratory syndrome coronavirus 2 (SARS-CoV-2) is a unique 2019 (COVID-19) deadly virus [1]. It is a highly contagious viral disease known to induce respiratory symptoms ranging from mild to severe pneumonia [2]. It has also been linked to a wide range of fungal and bacterial co-infections. A remarkable increase in cases of rhino-orbito-cerebral mucormycosis (ROCM) in COVID-19 patients has been reported [3,4,5], which is attributed to the combined effect of lung damage, immune modulation by therapies with broad-spectrum antibiotics, steroids, and antimicrobials, and a predilection for superinfections [6]. As a result of pandemic challenges, including lockdown, insufficient exercise, mental and emotional stress, and limited medical access, the majority of the population has shown increased glucose dysregulation. The activity of phagocytes is impaired by hyperglycaemia and acidosis, which constitute the primary host defense mechanism against fungal infections such as mucormycosis [7]. Hence, uncontrolled diabetes mellitus is a unique risk factor for ROCM. In COVID-19 patients, ROCM is associated with a high mortality rate, necessitating prompt treatment [6]. ROCM is an uncommon invasive fungal infection of the group Phycomycetes which attacks immunosuppressed hosts. It originates in the sinonasal mucosa and quickly extends to the orbit and the brain. Extensive angioinvasion is the leading cause of vascular thrombosis and tissue necrosis [8]. Infiltration of the cavernous sinus and orbital apex, with resulting facial cellulitis and vision loss, is a frequent complication with fatal consequences. Intracranial involvement can cause internal carotid artery (ICA) occlusion and ischemic infarctions [9].

Imaging plays an important role in the diagnosis and management of ROCM. Imaging usually helps in early disease identification, tracking the extent of the disease, accurate presurgical extent mapping, and post-treatment follow-up to determine the effectiveness of medical treatment and the sufficiency of surgical debridement [10]. Magnetic resonance imaging (MRI) is the imaging modality of choice for the detection of orbital and skull base infiltration, intracranial invasion, vascular blockage, and perineural dissemination. Due to the iron and manganese in the fungal components, MRI displays variable signal intensity in accordance with the sinus content [11]. T2 sluggish flow can be a sign of fungal invasion of the internal carotid artery [9].

This study reported the typical and rare MRI findings of histopathologically proven ROCM infections in COVID-19-positive patients.

## 2. Methods

### 2.1. Ethical Consideration

This study was authorized by the institutional review board (approval No: ZU-IRB# 8042, approved on 24 October 2021). The requirement for patient consent was waived. The study was done according to the code of ethics of the world medical association (Declaration of Helsinki) for studies involving humans.

### 2.2. Study Design and Population

Between October 2021 and December 2022, our hospital database was searched for all patients referred to the otolaryngology department for an MRI examination of the paranasal sinuses (PNS) due to clinically suspected mucormycosis infection (blood-tinged nasal discharge, periorbital swelling, facial pain, black turbinate, skin induration, and discoloration) after COVID-19 infection. The study included all patients with pathologically confirmed ROCM after COVID-19 infection and submitted to an MRI examination of the PNS. Patients who refused surgical intervention, lacked histopathological confirmation, and had poor-quality MRI images were excluded from the study.

### 2.3. MRI Image Acquisition

All MRI examinations were performed using a 1.5-T superconducting MR imager (Philips Achieva, Amsterdam, The Netherlands) with standard head and neck coils. The paranasal MRI protocol included axial T1WI fast spin echo images (TR/TE: 400/12 ms; FOV: 230 × 199, matrix: 232 × 179, gap: 0.4 mm), axial T2WI fast spin echo images (TR/TE: 4000/100 ms; FOV: 230 × 198, matrix: 288 × 210, gap: 0.4 mm), and axial, sagittal, and coronal post-contrast T1WI with and without fat-suppression (TR/TE: 570/20 ms, FOV:252 × 226, matrix: 280 × 197, gap: 0.4 mm) after intravenous injection of 0.1 mmol/kg of gadopentetate dimeglumine (2 mL/sec). MRI examination of the brain was performed in some patients and included axial T1WI, T2WI, axial fluid attenuation inversion recovery (FLAIR), axial diffusion-weighted (DW) images at b-values of 0 and 1000, and axial, sagittal, and coronal post-contrast T1WI.

### 2.4. MRI Image Interpretation

The MRI images were reviewed using a Picture Archiving and Communication System (PACS). Two experienced radiologists (with over 10 years of experience in MRIs of the head and neck) reviewed all images in consensus. They focused on the involvement of the sinuses, orbits, and intracranial spaces. In each case, plain T1WI and T2WI were reviewed. Sinuses with opacification were documented. The type of contrast enhancement, as well as the involvement of any extrasinus regions such as the orbit, masticator space, pterygopalatine fossa, face, cavernous sinus, and brain, were reported on a postcontrast MRI. Any complications, such as thrombosis of the arteries were recorded. Retroantral, masticator, orbital, and pterygopalatine involvement was identified by the presence of intrasinus soft tissue and fat stranding. Orbital cellulitis manifested as stranding in the retrobulbar fat with no obvious abscess formation. On post-contrast scans, the affected cavernous sinus and internal carotid artery displayed thickening, non-enhancement, and aberrant soft tissue. Extradural collections, dural enhancement, cerebritis, intracerebral abscesses, and infarcts were assessed in patients with intracranial extension. The “black turbinate” sign (focal regions of non-enhancing mucosa) was noted on contrast-enhanced MRI.

### 2.5. Histopathological Evaluation

Specimens were taken from the nasal cavity and/or PNS and stained with routine hematoxylin and eosin [H and E] for diagnosis. The diagnosis was validated using periodic acid-Schiff (PAS) to detect fungal hyphae, Masson’s trichrome to detect vascular changes, and a Caspase-3 immunohistochemical marker to detect apoptosis. Pathologists interpreted histopathological alterations such as necrosis, inflammation, and angioinvasion.

### 2.6. Treatment

The majority of treatments continue to involve systemic amphotericin B combined with surgical sinus debridement, ocular exenterations, and diabetes management.

### 2.7. Statistical Analysis

Data from recruited patients were expressed as means, standard deviations (SD), ranges, or numbers and percentages. Statistical analyses were accomplished using SPSS version 23. Statistical significance was set at *p* < 0.05.

## 3. Results

### 3.1. Demographic Data and Clinical Presentation

A total of 52 patients (30 (57.7%) males and 22 (42.3%) females; age range: 28–71 years; mean age 58 ± 11.8 years) had pathologically confirmed ROCM after COVID-19 infection. The demographic and clinical characteristics of the study patients are presented in Table 1. Most patients (44/52, 84.6%) were aged >40 years old. Patients aged 40–60 years were the most affected (29/52, 55.8%). At the time of imaging, all patients were admitted to the hospital for COVID-19. The most common clinical presentations at admission were dark-colored nasal discharge and/or nasal stuffiness (40/52, 76.9%), and facial swelling (34/52, 65.4%). Thirty-nine (75%) patients had an uncontrolled diabetic history, and eight (15.4%) received chemotherapy. All patients had been on oral corticosteroids from the diagnosis of COVID-19 until the time of admission. COVID-19 pneumonia necessitated mechanical ventilation in 28 (53.8%) patients. Injectable corticosteroids were administered to all patients (10–14 days). There was no evidence of lung fungal infection in any patient at the time of admission. In the ICU, all ROCM patients were treated by IV amphotericin B. Thirty-one (59.6%) patients underwent surgical debridement. Nineteen (36.5%) patients died during the study period. MRI examinations were performed with intravenous (IV) contrast in 45 (86.5%) patients and seven (13.5%) non-contrast examinations due to impaired renal function. Seven (13.5%) patients showed positive intracranial findings. The mean time interval between COVID-19 and ROCM was 17.3 ± 4.8 days (range: 8−39 days).

### 3.2. Sinonasal Involvement

In our study, the maxillary sinus was the most commonly affected PNS (50/52, 96.2%). In the majority of patients (30/52, 57.7%), multiple sinuses were involved. Unilateral sinus affection was more common (40/52, 76.9%) than bilateral sinus affection (12/52, 23.1%). Table 2 lists the percentage of affected sinuses in the study group.

### 3.3. MRI Findings and Signal Characteristics

On T1WI, all lesions were isointense or mildly hypointense. On T2WI, the lesions were heterogeneous in 19 (36.5%) patients, hyperintense in 18 (34.6%), and isointense to mildly hypointense in 15 (28.8%). Forty-seven (90.4%) patients showed restricted diffusion. The pattern of extension of infection and enhancement of the lesions were best demonstrated on fat-suppressed post-gadolinium images. Forty-five (86.5%) patients underwent contrast imaging. The patterns of enhancement seen in the post-contrast examination were as follows: nine (17.3%) patients showed intense homogeneous enhancement; 19 (36.5%) patients showed heterogeneous enhancement with variable enhancing and non-enhancing areas with a soap bubble appearance sign; and 17 (32.7%) patients showed complete central non-enhancement of the lesion with or without a thin irregular rim of peripheral enhancement. On contrast-enhanced images, enhancing soft tissue obliterating the retroantral fat was observed in 18 (34.6%) patients. The black turbinate sign was noted in 30 (57.7%) patients.

### 3.4. Extrasinus Extension

Forty-three (82.7%) patients had orbital involvement. Seven (13.5%) patients exhibited optic nerve affection and 11 (21.2%) showed extraocular muscle affection. The pterygopalatine fossa was involved in four (7.7%) patients. Three (5.8%) patients had cavernous sinus extension, two (3.8%) patients had pachymeningeal enhancement, and one (1.9%) patient had epidural collection. The alveolar margin was affected in two (3.8%) patients and there were extensions to the cheek in five (9.6%) patients. Based on the classification by Rupa et al. [12], most patients (29/52, 55.8%) appeared to have stage 2 disease at presentation. Additional details regarding the extension type are presented in Table 3.

### 3.5. Histopathological Findings

Regarding histopathological changes, granulomas were significantly more frequent in stage I (*p* = 0.0003), but angioinvasion was mostly found in stage III, as shown in Table 4.

**Figure 1:**
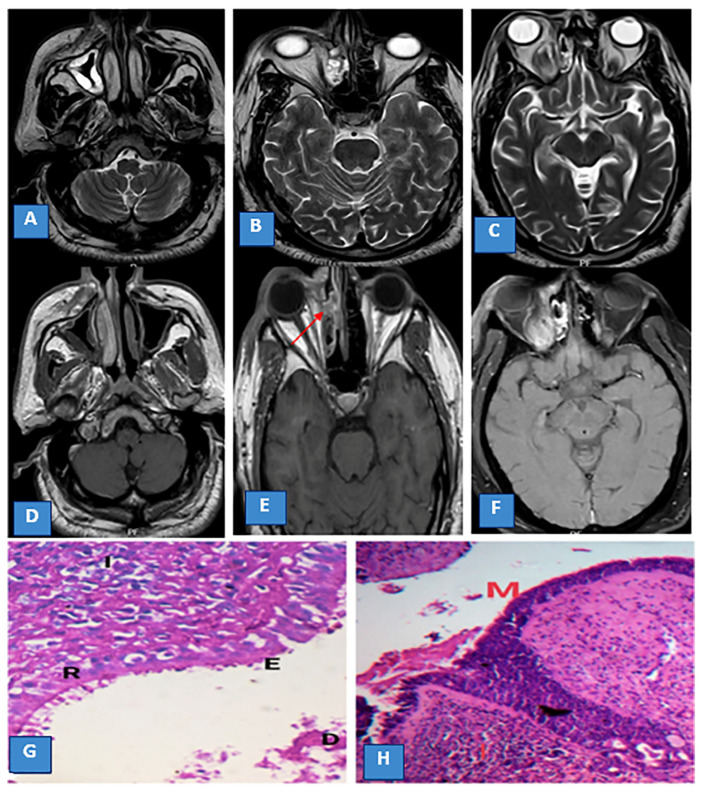
(**A**,**B**) Axial T2WI shows partial opacification of the right maxillary and ethmoid sinuses. (**C**) Axial T2WI shows extension of the inflammatory process to the apex of the right orbit with right eye proptosis. (**D**,**E**) Axial T1W postcontrast images show no significant post-contrast enhancement (black turbinate sign) (red arrow). (**F**) Axial T1W postcontrast fat suppression image shows enhancement of the right orbital apex. (**G**) Histopathological image of stage I nasal mucormycosis with detached epithelial cells (D) and erosion (E) of the respiratory mucosa (R) with underlying inflammatory cell infiltration, mainly eosinophils (×400, (**H**,**E**). (**H**) Histopathological image of stage I mucormycosis-induced maxillary sinusitis with several fragments of fungal molds (M) in the mucosa, with underlying inflammatory cells forming granulomas of epithelioid cells and lymphocytes (I) (×400, (**H**,**E**).

**Figure 2:**
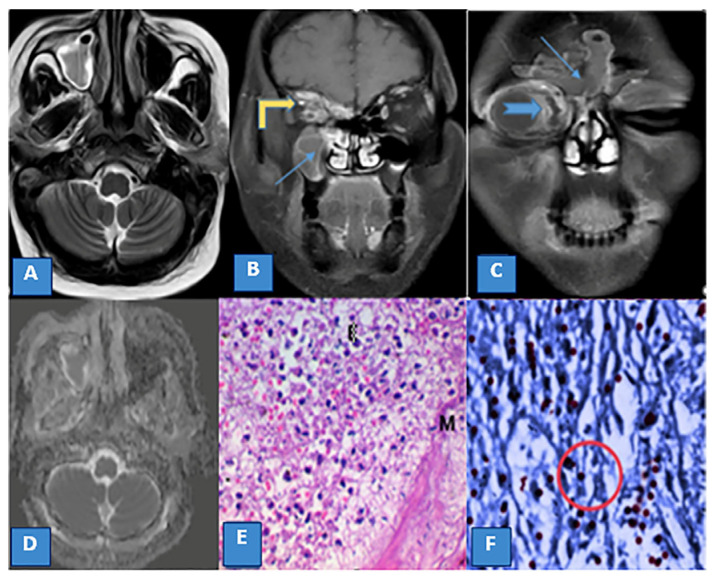
(**A**) Axial T2WI shows opacification of the right maxillary sinus with an intermediate signal. (**B**,**C**) Coronal T1 FAT suppressed postcontrast images show rim enhancement of the right maxillary and both frontal sinuses (thin blue arrows) with an extension of the inflammatory process to the medial wall of the right orbit (thick blue arrow) and around the optic nerve and orbital apex (yellow arrow). (**D**) ADC map showing restricted diffusion in the center of the right maxillary sinus. (**E**) Histopathological image of stage II mucormycosis-induced maxillary sinusitis with fungal hyphae (M) destroying the submucosa with extensive inflammatory cell infiltration, mainly eosinophils, admixed with hemorrhage and necrosis (×400, PAS). (**F**) Immunohistochemistry image of stage II mucormycosis-induced sinusitis with Positive brown cell for Caspases-3 denoting extensive apoptosis (red circle) (AI) (×400, Caspase-3).

**Figure 3:**
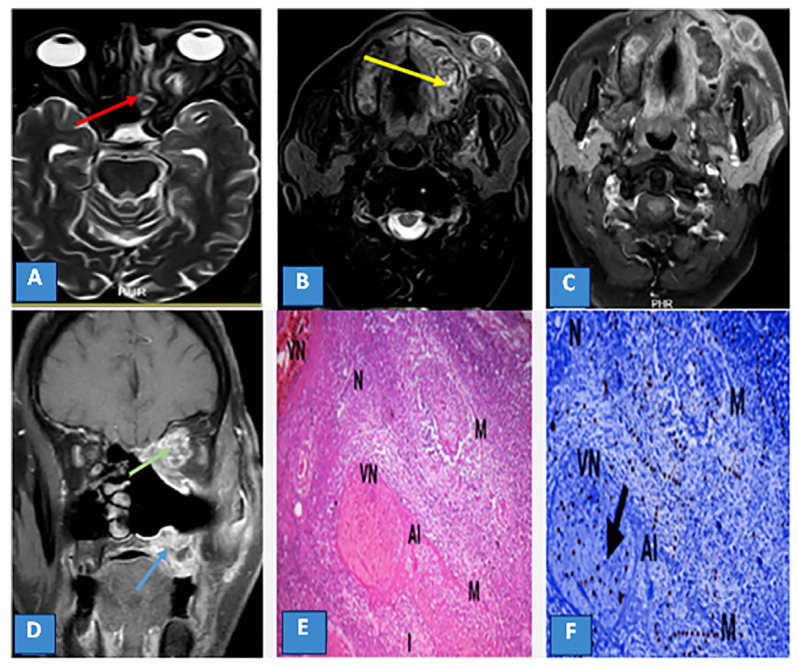
(**A**,**B**) Axial T2 STIR images show partial loss of pneumatization of the left ethmoid sinuses displaying intermediate to high signal (red arrow), with extension of inflammation to the left alveolar margin (yellow arrow) and subcutaneous region of the left cheek. (**C**) Axial T1 postcontrast fat-suppressed image shows heterogeneous enhancement of the lesion. (**D**) Coronal T1 postcontrast fat-suppressed image shows extensions of inflammation to the medial side of the left orbit with heterogeneous postcontrast enhancement (green arrow), and to the left alveolar margin (blue arrow). (**E**) Histopathological image of stage III mucormycosis-induced maxillary sinusitis with fungal hyphae (M) destroying the submucosa with inflammatory cells (I), necrosis (N) invading blood vessels denote angioinvasion and vascular necrosis (VN) with increasing thickness of the pale pink necrotic vessel wall (×400, Masson trichrome). (**F**) Immunohistochemistry image of stage II mucormycosis-induced sinusitis from the previous image with a positive brown cell for Caspases-3 (arrow) denoting extensive apoptosis (AI) (×400, Caspase-3).

**Figure 4:**
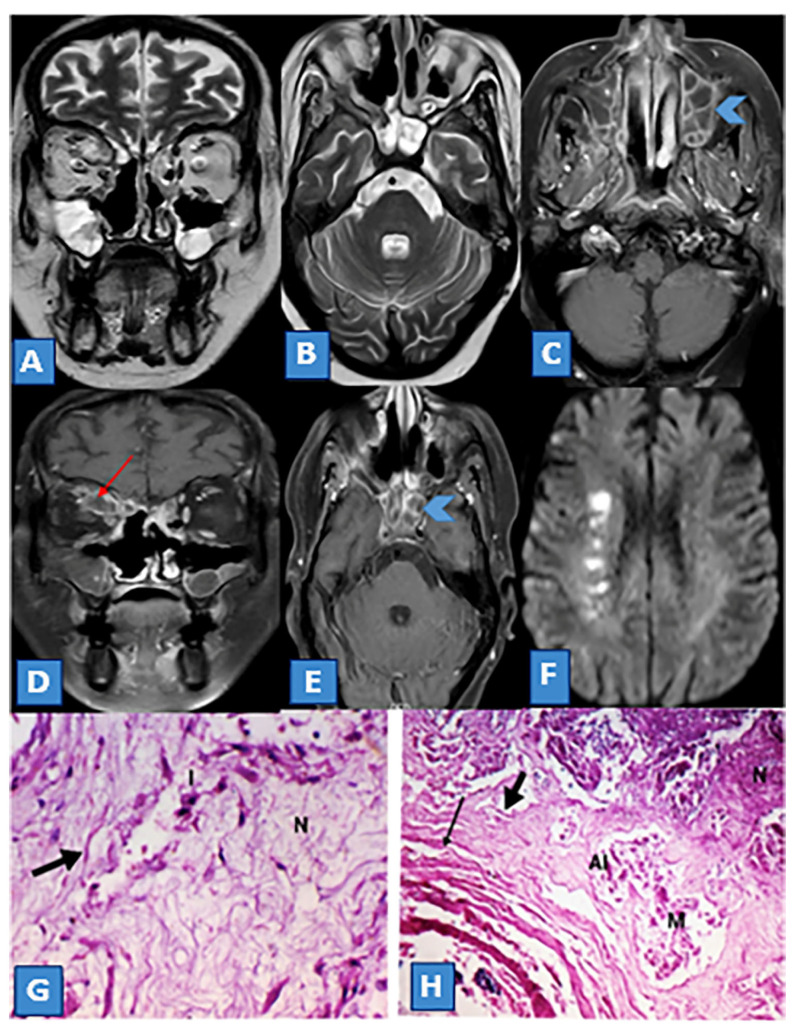
(**A**) Coronal and (**B**) Axial T2WI shows partial opacification of both the maxillary and sphenoid sinuses. (**C**,**E**) Axial T1 postcontrast fat-suppressed images show heterogeneous enhancement with enhanced septa (soap bubble sign) (arrowheads). (**D**) Coronal T1 postcontrast fat-suppressed image reveals an extension of the infection to the right orbit with a subperiosteal abscess (red arrow). (**F**) Axial brain DWI demonstrates multiple zones of restricted diffusion at the right cerebral internal border (a string of pearls), denoting a watershed infarction between the right middle cerebral artery and anterior cerebral artery territories. (**G**) Histopathology image of stage III mucormycosis-induced maxillary sinusitis showing fungal hyphae (arrow) destroying the tissue with inflammatory cells (I) and necrosis (N) (×400, PAS). (**H**) Fragments of fungal hyphae (M) (arrows) invading blood vessels, denoting angioinvasion (AI) and vascular necrosis (VN) with areas of necrosis (N) surrounding fungal hyphae (M) (×100, PAS).

Figure 1, Figure 2, Figure 3 and Figure 4 show the sample cases from our study.

## 4. Discussion

COVID-19 is linked to a high rate of secondary fungal and bacterial infections, mostly due to immunological dysregulation. Additionally, the extensive use of broad-spectrum antibiotics, monoclonal antibodies, and steroids as part of the COVID-19 treatment protocol may result in developing or worsening preexisting fungal illnesses [13,14,15]. Physicians should be aware of the likelihood of invasive secondary fungal infections in patients with COVID-19, particularly those with prior risk factors. They should be able to detect and treat these infections early, thus reducing mortality and morbidity rates. The usage of therapeutic agents should be closely monitored to obtain the best possible therapeutic effect at the lowest possible dose and for the shortest possible time. Broad-spectrum antibiotics should be reconsidered, especially in the absence of infections [16].

Mucormycosis is a rare opportunistic fungal infection that causes angioinvasive illnesses in affected tissues, resulting in severe necrosis and infarction. Orbits and PNS are affected by ROCM, which can spread to the brain parenchyma [17,18].

This study presents the MRI findings of 52 patients with confirmed ROCM in COVID-19. MRI is a valuable tool for diagnosing mucormycosis infections that affect the sinonasal area, orbits, and possible intracranial extension [19]. MRI’s multi-planar capabilities and better soft tissue visualization aid in determining the anatomical extent of the disease and associated sequelae [19,20].

Herrera et al. [19] agreed that opacification of the affected PNS and/or mucosal thickening are imaging findings of mucormycosis. On T1-weighted images, most lesions were hypointense, whereas T2WI showed variable hyperintense lesions. In agreement with our study, Safder et al. [21] stated that on T2WI, a low signal intensity of fungal components may be detected with restricted diffusion on DWI.

Nasal turbinate hypertrophy and nasal secretions are associated with nasal involvement [20]. The involved tissues and thickened mucosa showed postcontrast enhancement. The “black turbinate sign” describes regions of non-enhancing soft tissue inside the affected nasal turbinate and/or PNS [21,22]. This sign may aid in identifying nasal mucormycosis in its early stages [21]. Howells et al. [23] and Li et al. [24] previously documented the MR features of acute fulminant invasive fungal sinusitis (AFIFS) as follows: sinus opacification, air-fluid level, variable intensity within the sinuses on T1WI and T2WI (predominant hypointense on T2WI), the black turbinate sign (non-enhancing hypointense turbinate), preantral fat infiltration, obliteration of the nasopharyngeal planes, loss of contrast enhancement of the sinonasal mucosa and extraocular muscles, inflammatory changes in the extraocular fat and muscles, and cerebral pachymeningeal enhancement. The current study concluded that the imaging features of mucormycosis in post-COVID-19 patients did not vary from those previously documented in the AFIFS. Contrary to Gorovoy et al. [25], orbital extension, bone erosion, and retroantral fat pad inflammation were specific but less prevalent late findings of AFIFS. However, we reported that they were involved early in post-COVID-19 mucormycosis. In addition, according to Howells et al. [23], maxillofacial bone infiltration is always a late finding; however, in the current study, we noticed that maxillofacial bone infiltration was not always a late finding in our post-COVID-19 mucormycosis patients.

The extra-sinus spread into the orbit or the face is common and can extend into the infratemporal fossa, skull base, cavernous sinus, and intracranial compartment [20]. A follow-up MRI may be required for some patients after treatment.

Advanced MRI sequences such as susceptibility-weighted imaging (SWI) or quantitative susceptibility mapping (QSM) are very sensitive to the presence of paramagnetic materials in the brain and other tissues. Since ROCM as a fungal infection is associated with elevated levels of iron and manganese in affected tissues, these advanced MRI sequences can therefore be effective methods in the diagnosis of RCOM. However, we did not routinely use such sequences in our institution and depend mainly on the standard MRI protocol for diagnosis of ROCM.

Regarding the histopathology, Lue et al. [26] agreed that mucormycosis sections showed moderate chronic inflammation in the mucosa, associated with inflammatory exudates, granulation tissue proliferation, necrosis, and numerous hyphae of mucormycosis depending on the severity of the lesion. However, Ibrahim et al. [27] proposed an alternative explanation for mucormycosis infection, namely that the hallmark of mucormycosis infection is the uniform presence of extensive angioinvasion with tissue necrosis unrelated to the disease stage.

The study’s limitations included limited sample size, lack of evidence of a link between imaging results and clinical outcomes, and no major differences in results between ROCM in COVID-19 and ROCM in other scenarios.

## 5. Conclusions

In the context of COVID-19, radiologists should be aware of the increasing incidence of invasive ROCM and should search for the aforementioned imaging abnormalities to aid in early identification and diagnosis. Early diagnosis allows rapid antifungal treatment, which reduces mortality and morbidity. Imaging is essential for determining the degree and extent of the disease and planning surgical debridement. Removal of all necrotic tissues increased the survival rate.

## Figures and Tables

**Table 1:** Demographic and clinical presentations of the study population.

	Value
Age, years, mean SD (range)	58 ± 11.8 (28–71)
Sex	
Male	30 (57.7)
Female	22 (42.3)
Clinical presentations	
Facial pain and numbness	10 (19.2)
Facial swelling	34 (65.4)
Dark colored nasal discharge and/or nasal stuffiness	40 (76.9)
Epistaxis	7 (13.5)
Blurred vision	30 (57.7)
Proptosis	29 (55.8)
Ptosis	3 (5.8)
Ophthalmoplegia	19 (36.5)
Facial paresis	3 (5.8)
Stroke	2 (3.8)
Dental symptoms such as loosening of teeth or jaw pain	2 (3.8)
Chronic diseases	
Uncontrolled diabetic history	39 (75)
Type 2 diabetes mellitus	34 (65.4)
Type 1 diabetes mellitus	5 (9.6)
Hematological disorders	3 (5.8)
HIV	2 (3.8)
Autoimmune diseases	7 (13.5)
Received chemotherapy	8 (15.4%)
Mechanical ventilation	
Yes	28 (53.8)
No	24 (46.2)
Treatment	
Injectable corticosteroids	52 (100)
IV amphotericin B	52 (100)
underwent surgical debridement.	31 (59.6)
Fatality	
Survived	33 (63.5)
Died	19 (36.5)

Unless otherwise indicated, the data represent the number of cases with percentages in parentheses. SD: standard deviation.

**Table 2:** The involved sinuses in post-COVID-19 mucormycosis patients.

Sinuses Involved	Numbers	%
Frontal	5	9.6
Maxillary	50	96.2
Ethmoid	37	71.2
Sphenoid	26	50
Pansinusitis	3	5.8

**Table 3:** Extent of regional involvement.

Stage	Involved Areas	Numbers	%
Stage 1	Nose and paranasal sinuses alone	7	13.5
Stage 2	Paranasal sinuses with immediate adjacent areas which are surgically resectable with minimal morbidity e.g., orbit (extra-conal), oral cavity and palate	29	55.8
Stage 3	Intracranial extension (extradural/intra-cerebral) or partially resectable with extension to pterygopalatine fossa, cavernous sinus, periorbital region and cheek	16	30.8

**Table 4:** Histopathological changes in our post-COVID mucormycosis patients.

Variable	Stage I *n* = 7	Stage II *n* = 29	Stage III *n* = 16	Total *n* = 52
Severe Inflammation	1 (14.3)	4 (13.8)	2 (12.5)	7 (13.5)
Granuloma	4 (42.9)	1(3.5)	0 (0)	5 (9.6)
Angioinvasion	1 (14.3)	1 (3.5)	6 (37.5)	8 (15.4)
Positive Apoptosis marker (Caspase-3)	1 (14.3)	23 (79.3)	8 (50)	32 (61.5)

Data represent the number of cases with percentages in parentheses.

## Data Availability

The datasets used and/or analyzed during the current study are available from the corresponding author upon reasonable request.

## References

[1] World Health Organization Coronavirus. https://www.who.int/health-topics/coronavirus#tab=ta.

[2] Kwee T.C., Kwee R.M. (2020). Chest CT in COVID-19: What the Radiologist Needs to Know. Radiographics.

[3] Revannavar S.M., Supriya P.S., Samaga L., Vineeth V. (2021). COVID-19 triggering mucormycosis in a susceptible patient: A new phenomenon in the developing world?. BMJ Case Rep..

[4] El-Kholy N.A., El-Fattah A.M.A., Khafagy Y.W. (2021). Invasive Fungal Sinusitis in Post COVID-19 Patients: A New Clinical Entity. Laryngoscope.

[5] Ismaiel W.F., Abdelazim M.H., Eldsoky I., Ibrahim A.A., Alsobky M.E., Zafan E., Hasan A. (2021). The impact of COVID-19 outbreak on the incidence of acute invasive fungal rhinosinusitis. Am. J. Otolaryngol..

[6] Bhatt K., Agolli A., Patel M.H., Garimella R., Devi M., Garcia E., Amin H., Domingue C., Guerra Del Castillo R., Sanchez-Gonzalez M. (2021). High mortality co-infections of COVID-19 patients: Mucormycosis and other fungal infections. Discoveries.

[7] Sekaran A., Patil N., Sabhapandit S., Sistla S.K., Reddy D.N. (2022). Rhino-orbito-cerebral mucormycosis: An epidemic in a pandemic. IJID Reg..

[8] Gamaletsou M.N., Sipsas N.V., Roilides E., Walsh T.J. (2012). Rhino-orbital-cerebral mucormycosis. Curr. Infect. Dis. Rep..

[9] Lone P.A., Wani N.A., Jehangir M. (2015). Rhino-orbito-cerebral mucormycosis: Magnetic resonance imaging. Indian J. Otol..

[10] Groppo E.R., El-Sayed I.H., Aiken A.H., Glastonbury C.M. (2011). Computed tomography and magnetic resonance imaging characteristics of acute invasive fungal sinusitis. Arch. Otolaryngol.–Head Neck Surg..

[11] Parsi K., Itgampalli R.K., Vittal R., Kumar A. (2013). Perineural spread of rhino-orbitocerebral mucormycosis caused by Apophysomyces elegans. Ann. Indian Acad. Neurol..

[12] Rupa V., Maheswaran S., Ebenezer J., Mathews S.S. (2015). Current therapeutic protocols for chronic granulomatous fungal sinusitis. Rhinology.

[13] Shah V.K., Firmal P., Alam A., Ganguly D., Chattopadhyay S. (2020). Overview of immune response during SARS-CoV-2 infection: Lessons from the past. Front. Immunol..

[14] Mekonnen Z.K., Ashraf D.C., Jankowski T., Grob S.R., Vagefi M.R., Kersten R.C., Simko J.P., Winn B.J. (2021). Acute invasive rhino-orbital mucormycosis in a patient with COVID-19-associated acute respiratory distress syndrome. Ophthalmic Plast. Reconstruct. Surg..

[15] Sonkar C., Hase V., Banerjee D., Kumar A., Kumar R., Jha H.C. (2022). Post COVID-19 complications, adjunct therapy explored, and steroidal after effects. Can. J. Chem..

[16] Mehta S., Pandey A. (2020). Rhino-Orbital Mucormycosis Associated With COVID-19. Cureus.

[17] Ferguson B.J. (2000). Mucormycosis of the nose and paranasal sinuses. Otolaryngol. Clin. N. Am..

[18] Werthman-Ehrenreich A. (2021). Mucormycosis with orbital compartment syndrome in a patient with COVID-19. Am. J. Emerg. Med..

[19] Herrera D.A., Dublin A.B., Ormsby E., Aminpour S., Howell L.P. (2008). Imaging Findings of Rhinocerebral Mucormycosis. Skull Base.

[20] Therakathu J., Prabhu S., Irodi A., Sudhakar S.V., Yadav V.K., Rupa V. (2018). Imaging features of rhinocerebral mucormycosis: A study of 43 patients. Egypt. J. Radiol. Nucl. Med..

[21] Safder S., Carpenter J.S., Roberts T.D., Bailey N. (2010). The “Black Turbinate” sign: An early MR imaging finding of nasal mucormycosis. AJNR Am. J. Neuroradiol..

[22] Taylor A.M., Vasan K., Wong E.H., Singh N., Smith M., Riffat F., Sritharan N. (2020). Black Turbinate sign: MRI finding in acute invasive fungal sinusitis. Otolaryngol. Case Rep..

[23] Howells R.C., Ramadan H.H. (2001). Usefulness of computed tomography and magnetic resonance in fulminant invasive fungal rhinosinusitis. Am. J. Rhinol..

[24] Li Z., Wang X., Jiang H., Qu X., Wang C., Chen X., Chong V.F., Zhang L., Xian J. (2020). Chronic invasive fungal rhinosinusitis vs sinonasal squamous cell carcinoma: The differentiating value of MRI. Eur. Radiol..

[25] Gorovoy I.R., Kazanjian M., Kersten R.C., Kim H.J., Vagefi M.R. (2012). Fungal rhinosinusitis and imaging modalities. Saudi J. Ophthalmol..

[26] Luo L.C., Cheng D.Y., Zhu H., Shu X., Chen W.B. (2009). Inflammatory pseudotumoural endotracheal mucormycosis with cartilage damage. Eur. Respir. Rev..

[27] Ibrahim A.S., Spellberg B., Avanessian V., Fu Y., Edwards J.E. (2005). Rhizopus oryzae adheres to, is phagocytosed by, and damages endothelial cells in vitro. Infect. Immun..

